# Extracellular ATP reduces HIV-1 transfer from immature dendritic cells to CD4^+ ^T lymphocytes

**DOI:** 10.1186/1742-4690-5-30

**Published:** 2008-03-28

**Authors:** Corinne Barat, Caroline Gilbert, Michael Imbeault, Michel J Tremblay

**Affiliations:** 1 Laboratoire d'Immuno-Rétrovirologie Humaine, Centre de Recherche en Infectiologie, RC709, 2705 Boul. Laurier, Québec (QC), G1V 4G2, Canada

## Abstract

**Background:**

Dendritic cells (DCs) are considered as key mediators of the early events in human immunodeficiency virus type 1 (HIV-1) infection at mucosal sites. Previous studies have shown that surface-bound virions and/or internalized viruses found in endocytic vacuoles of DCs are efficiently transferred to CD4^+ ^T cells. Extracellular adenosine triphosphate (ATP) either secreted or released from necrotic cells induces a distorted maturation of DCs, transiently increases their endocytic capacity and affects their migratory capacity. Knowing that high extracellular ATP concentrations are present in situations of tissue injury and inflammation, we investigated the effect of ATP on HIV-1 transmission from DCs to CD4^+ ^T lymphocytes.

**Results:**

In this study, we show that extracellular ATP reduces HIV-1 transfer from immature monocyte-derived DCs (iDCs) to autologous CD4^+ ^T cells. This observed decrease in viral replication was related to a lower proportion of infected CD4^+ ^T cells following transfer, and was seen with both X4- and R5-tropic isolates of HIV-1. Extracellular ATP had no effect on direct CD4^+ ^T cell infection as well as on productive HIV-1 infection of iDCs. These observations indicate that extracellular ATP affects HIV-1 infection of CD4^+ ^T cells *in trans *with no effect on *de novo *virus production by iDCs. Additional experiments suggest that extracellular ATP might modulate the trafficking pathway of internalized virions within iDCs leading to an increased lysosomal degradation, which could be partly responsible for the decreased HIV-1 transmission.

**Conclusion:**

These results suggest that extracellular ATP can act as a factor controlling HIV-1 propagation.

## Background

Dendritic cells (DCs) are crucial in generating a virus-specific immune response since they are recognized as the most potent antigen-presenting cells of the immune system, yet they also play a pivotal role in establishment and dissemination of HIV-1 infection. Indeed, HIV-1 exploits the migratory ability of DCs to gain access to CD4^+ ^T lymphocytes in lymphoid tissues. DCs, particularly immature DCs (iDCs) that reside in peripheral mucosal tissues, are among the first target cells for the virus. The reported low levels of CD4 and chemokine co-receptors CXCR4 and CCR5 on DCs are probably responsible for their weaker susceptibility to productive HIV-1 infection *in vitro *as compared to CD4^+ ^T cells [1,2]. It has been demonstrated that interactions between the external HIV-1 envelope and C-type lectin receptors such as DC-specific intercellular adhesion molecule 3-grabbing nonintegrin (DC-SIGN) [3,4], mannose receptor (MR) (CD206) and galactosyl ceramide [5] result in an efficient capture of viral particles. Following interactions with the viral entity, DCs then migrate to lymphoid tissues where their contact with CD4^+ ^T cells promotes a highly potent viral transfer, resulting in vigorous HIV-1 replication. Although viruses can remain either surface-bound or be taken up into endolysosomal vacuoles, the origin of the transferred virus is still a matter of debate and could be different in iDCs versus mature DCs (mDCs) [6-10]. Interestingly, HIV-1 transfer from DCs to CD4^+ ^T cells was shown to occur in two phases [11], which are not necessarily sequential or interdependent [12]. In the initial transfer phase (i.e. early transfer or *trans*-infection) the virus located within DCs rapidly relocate at the DC/T-cell contact zone, this locally virus-concentrated region between the two cell types being referred to as the virological synapse [9,13]. The second phase (i.e. late transfer or *cis*-infection) is dependent on productive infection of DCs and eventual transfer of progeny virus to CD4^+ ^T cells [14]. A viral particle encountering an iDC can have different fates. Entry using CD4/co-receptor complexes will lead to viral replication and transfer of progeny virus. On the other hand, after capture by a DC-SIGN-mediated endocytosis process, the majority of virus particles will be degraded inside endolysosomal compartments followed by an antigenic presentation in the context of major histocompatibility complex class II molecules. A recent report has demonstrated that an interaction between DC-SIGN and leukocyte-specific protein A helps to shuttle HIV-1 particles to the proteasome, thereby promoting their eventual degradation [15]. A small fraction of such endocytosed virions will escape processing and retain their infectivity. These viruses may be released to the cytoplasm, leading to DC infection [16], or remain protected from degradation in a low pH compartment lacking early or late endosomal markers and are later relocated to the virological synapse [9,13,17]. The molecular mechanisms governing HIV-1 storage and transfer by DCs are still poorly understood. For example, the signals leading to the formation of the virological synapse have yet to be identified. Also, little is known about the contribution of intracellular signal transducers in virus capture and transfer, although it has been recently demonstrated that Src and Syk tyrosine kinases play a functional role in productive HIV-1 infection of iDCs [18].

Extracellular nucleotides have emerged as important regulators of the immune response [19]. Adenosine 5'-triphosphate (ATP) is present at high concentrations in the cytosol and intracellular compartments, and can be secreted by many cell types including epithelial and endothelial cells, platelets, macrophages and activated T lymphocytes [20]. In addition, ATP is released in large amounts upon plasma membrane damage or necrotic cell death. Hence, ATP functions as an indicator of tissue destruction for the immune system. It has been demonstrated that ATP modulates the function of immune cells by activating two groups of purinergic receptors: P2X receptors, which are ligand-gated cation channels, and the seven membrane-spanning P2Y receptors, which are coupled to G proteins [19]. An ATP exposure induces mostly a proinflammatory response and it is now considered as a danger signal to the immune system. Expression of both receptor types has been reported in DCs [21], and extracellular ATP exerts many effects on DC functions. In monocyte-derived DCs, ATP was shown to induce a distorted maturation state, with the up-regulation of costimulatory and adhesion molecules (e.g. CD54, CD80, CD83 and CD86), an increased capacity to migrate to lymph nodes associated with a modulation of the expression of various chemokines and chemokine receptors, and a reduced ability to attract Th1-type lymphocytes [22,23]. In addition, ATP inhibits LPS- and CD40L-induced production of interleukin (IL)-1α and β, tumor necrosis factor (TNF)-α, IL-6 and IL-12 [22,24], but synergize with TNF-α in the activation of DCs [25]. More recently, ATP was shown to up-regulate two genes in DCs that are involved in immunosuppression, i.e. thrombospondin-1 and indoleamine 2,3-dioxygenase [26]. At high concentrations, ATP induces profound morphological changes and apoptotic cell death [27]. Interestingly, ATP affects also the endocytic activity [22,25] and induces actin reorganization [28] in human DCs.

Knowing that an increase in extracellular ATP concentrations is seen in situations of tissue injury, inflammation or microbial invasion, we investigated the effect of ATP on HIV-1 transfer from DCs to CD4^+ ^T lymphocytes. Here we show that a short exposure to extracellular ATP causes a decrease in viral transfer from iDCs to autologous CD4^+ ^T cells. Additional studies indicate that ATP modulates the first phase of virus transfer, whereas the late transfer step and *de novo *infection of iDCs are not affected. Our results suggest that ATP exerts its effects partly by promoting degradation of HIV-1 within iDCs. These results suggest that extracellular ATP, which can be released under proinflammatory conditions, does not only act as a danger signal for the immune system but also as a factor controlling HIV-1 transmission.

## Results

### Extracellular ATP diminishes HIV-1 transfer from DCs to CD4^+ ^T cells

**Figure 1:**
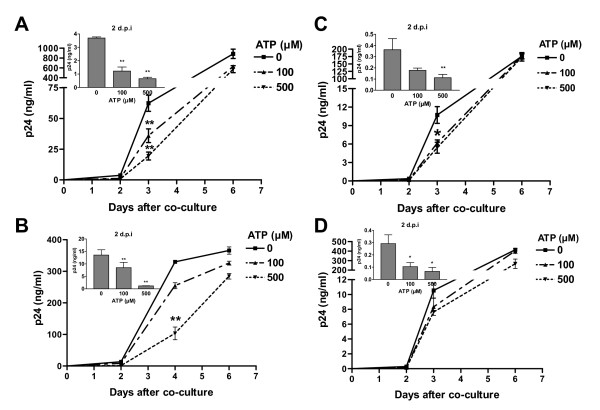
**Extracellular ATP reduces HIV-1 transfer from iDCs to autologous CD4^+ ^T cells**. iDCs were initially pulsed with R5-tropic NL4-3Bal*env *either produced upon transient transfection of 293T cells (A) or following acute infection of CD4^+ ^T cells (B). In some experiments, iDCs were pulsed with X4-tropic viruses consisting of the laboratory variant NL4-3 produced upon transfection of 293T cells (C) or the clinical strain 92HT599 amplified in PBMCs (D). Pulsing was performed in absence or presence of the indicated concentrations of ATP for 60 min at 37°C. Next, iDCs were washed extensively to remove input virus and excess ATP and co-cultured with autologous CD4^+ ^T cells at a 1:3 ratio. Virus production was assessed by measuring the cell-free p24 contents at the listed time points. Virus production at day 2 following initiation of the co-culture is depicted in the small inserts (upper left part of each panel). The data shown represent the means ± standard deviations of triplicate samples and are representative of four independent experiments for panels A, C and D, and three for panel B. Statistical analysis was performed on the results from all experiments. Asterisks denote statistically significant data (*, P < 0.05; **, P < 0.01).

**Figure 2:**
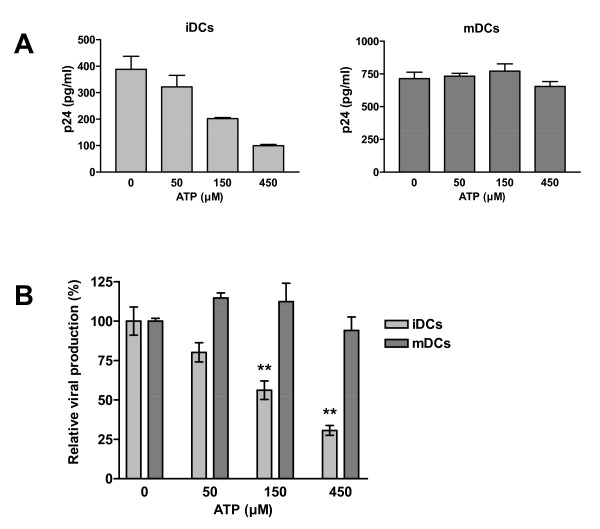
**ATP affects virus transfer from iDCs but not mDCs**. iDCs and mDCs from the same donor were first pulsed for 60 min at 37°C with R5-tropic JR-CSF either in absence or presence of ATP at the listed concentrations. Next, cells were washed extensively to remove input virus and co-cultured with autologous CD4^+ ^T lymphocytes at a 1:3 ratio. Viral production was assessed at day 2 following initiation of the co-culture. Results from one representative experiment with triplicate samples are presented in panel A. The data shown in panel B represent the means ± standard deviations calculated from two independent experiments and are expressed as relative viral production compared to untreated cells. Asterisks denote statistically significant data (**, P < 0.01).

To study the possible consequences of ATP on HIV-1 capture by DCs and subsequent virus transfer to CD4^+ ^T cells, iDCs were first pulsed with R5-tropic NL4-3Bal*env *produced in 293T cells in the presence of ATP. Next, iDCs were co-cultured with autologous CD4^+ ^T cells without any exogenous ATP. Data from studies using this experimental design indicate that HIV-1 transfer is reduced by the two concentrations of ATP tested (i.e. 100 and 500 μM) (Fig. 1A). Higher concentrations of ATP were found to induce apoptosis in iDCs (i.e. >500 μM) (data not shown) and were not used in the following experiments. The fact that the observed inhibition is more significant at earlier time points following initiation of the co-culture is indicative of an ATP-dependent effect on interactions between HIV-1 and iDCs. Indeed, virus infection is spreading to the CD4^+ ^T-cell population at later time points and the ATP-directed modification of virus production decreased with time. Similar observations were made when using different virus preparations such as NL4-3Bal*env *amplified in CD4^+ ^T cells (Fig. 1B) or X4-tropic viruses such as NL4-3 produced in 293T cells (Fig. 1C) and the clinical isolate 92HT599 amplified in peripheral blood mononuclear cells (PBMCs) (Fig. 1D). Altogether data from these studies demonstrate that the ATP-induced effect on HIV-1 transfer is not influenced by the viral isolate, tropism, or virus producer cell. The ATP-dependent effect on virus propagation was found to be dose-dependent when using iDCs as transmitter cells (Fig. 2a, left panel). Although the maturation process of DCs results, as expected, to a more significant virus transmission, the ATP-mediated decrease in HIV-1 transfer is no longer seen with mDCs (Fig. 2a&b, compare left and right panels). Therefore, the next series of investigations were performed with iDCs only.

### HIV-1 is transferred to a smaller number of CD4^+ ^T cells in the presence of ATP

**Figure 3:**
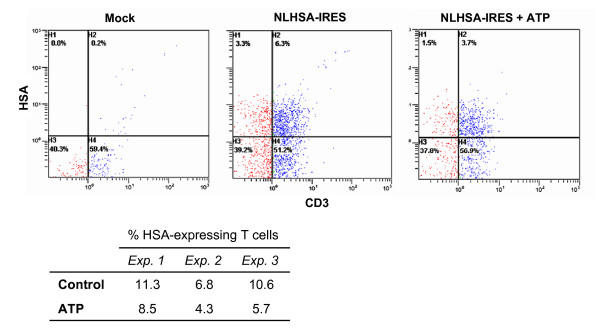
**HIV-1 is transferred to a lower number of CD4^+ ^T cells in presence of ATP**. iDCs were either left untreated (mock) or pulsed for 60 min at 37°C with NLHSA-IRES (20 ng of p24/10^5 ^cells) in absence or presence of ATP (400 μM). Next, cells were extensively washed to remove input virus and co-cultured for 3 days with autologous CD4^+ ^T cells. Cells were then stained for CD3 (FITC labeling) and HSA (R-PE labeling) and analyzed by flow cytometry. The percentage of HSA-expressing cells in the CD3-positive population is shown for three independent experiments.

To define if the observed ATP-driven decrease in virus production in co-cultures made of iDCs and autologous CD4^+ ^T cells was caused by a reduced number of HIV-1-infected CD4^+ ^T cells, we used the NLHSA-IRES molecular construct that we have recently engineered (Imbeault *et al*., manuscript in preparation). This X4-tropic infectious molecular clone of HIV-1 codes for all viral genes and also for a cell surface reporter molecule, i.e. the murine heat-stable antigen (HSA). Unlike most of the previous reporter constructs, this plasmid will lead to the production of fully infectious virions with no deletions in *env*, *vpr *or *nef*, and allow the detection of productively infected cells through the surface expression of the HSA molecule. The pulsing of iDCs with such viruses followed by a co-culture step with autologous CD4^+ ^T cells for 3 days resulted in a proportion of CD4^+ ^T lymphocytes productively infected with HIV-1 ranging from 6.8 to 11.3% as monitored by measuring the percentage of cells positive for both HSA and CD3. This population of double positive cells was reduced by 25 to 46% when ATP was present during the pulsing step. Flow cytometry data from a representative experiment are depicted in Fig. 3. This observation suggests that treatment of iDCs with ATP leads to a decrease in the number of CD4^+ ^T cells productively infected with HIV-1 following transfer of the virus by iDCs.

### ATP affects the early phase of transfer

**Figure 4:**
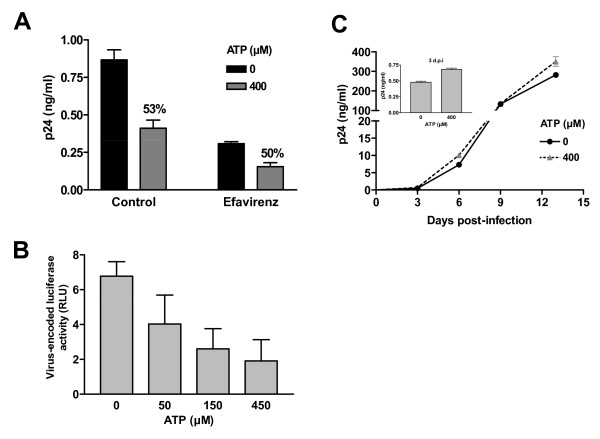
**Extracellular ATP affects the early transfer phase**. (A) iDCs were initially either left untreated or treated with 50 nM efavirenz. Next, cells were either left untreated or treated with ATP before addition of NL4-3Bal*env*. Finally, cells were washed and co-cultured with autologous CD4^+ ^T cells for 2 days before assessing viral production. The percentage of inhibition is shown above the appropriate bars. (B) Transfer studies were carried out using recombinant luciferase-encoding viruses pseudotyped with the JR-FL envelope. Luciferase activity was assessed at 3 days following initiation of the co-culture. (C) iDCs were pulsed for 60 min with NL4-3Bal*env *in absence or presence of ATP (400 μM). Next, iDCs were extensively washed to remove input virus and cultured in complete culture RPMI medium supplemented with GM-CSF and IL-4. Cell-free supernatants were collected and assayed for the p24 content at the indicated days post-infection. Data shown represent the means ± standard deviations of triplicate samples and are representative of three (panels A and C) or two (panel B) independent experiments.

Previous reports have shown that HIV-1 is efficiently transferred from iDCs to CD4^+ ^T cells via a process involving two distinct phases [7,11,18,29]. An initial transfer phase occurs whereby intact viruses captured by iDCs are transported to the DC-T cell synapse and transferred to CD4+ T cells (i.e. early transfer or *trans*-infection). In a second, slower process, a productive infection of iDCs results in a potent transfer of newly formed viruses to CD4^+ ^T cells (i.e. late transfer or *cis*-infection). Knowing that the late transfer phase occurs almost exclusively with R5-tropic viruses and having shown that ATP affects equally the transfer of both R5 and X4 viruses, we made the hypothesis that the presence of ATP during DC pulsing affect only the early transfer phase. To confirm this hypothesis, iDCs were pretreated or not with the antiretroviral drug efavirenz before being used for HIV-1 transfer studies. This treatment, by preventing productive infection of iDCs, allows only the first transfer phase to take place. As expected, this treatment reduced the amount of transferred viruses (Fig. 4A). However a comparable ATP-mediated decrease in HIV-1 transmission was seen in both untreated and efavirenz-treated cells (i.e. 53 versus 50% inhibition, respectively). In addition, a similar decrease in virus transfer was seen when using a single-cycle reporter virus pseudotyped with a R5 envelope (Fig. 4B), therefore substantiating the idea that ATP is acting on the *trans*-infection process where the captured viruses are directed to the DC-T-cell synapse. To confirm that ATP modulates only this early transfer step, we analysed its effect on productive infection in iDCs. To this end, iDCs were pulsed with fully competent R5-using viruses (i.e. NL4-3Bal*env*) in absence or presence of ATP and *de novo *virus production was evaluated over time. Results illustrated in Fig. 4C indicate that ATP has no effect on productive HIV-1 infection of iDCs and thus corroborate that ATP is not affecting the second long-term transfer phase. Collectively, these data suggest that only the first phase of viral transfer is affected by ATP (i.e. *trans*-infection).

### ATP affects virus degradation within iDCs

**Figure 5:**
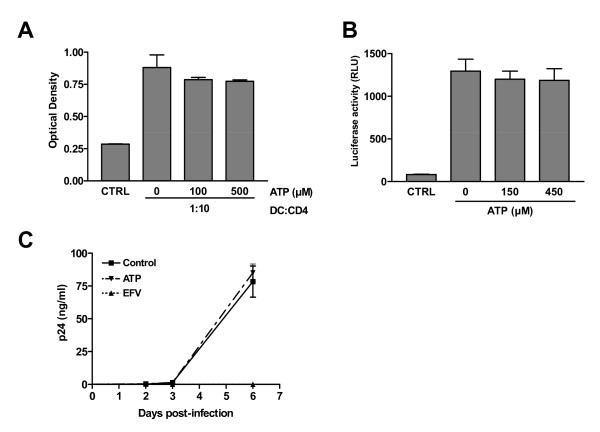
**ATP is neither affecting the T-cell stimulatory capacity of iDCs nor virus production in CD4^+ ^T cells**. (A) iDCs were either left untreated or treated for 60 min at 37°C with the indicated concentrations of ATP. Next, cells were extensively washed and co-cultured for 3 days with heterologous CD4^+ ^T cells at a 1:10 ratio. Cellular proliferation was finally assessed using a colorimetric MTS assay. (B) iDCs were either left untreated or treated for 60 min at 37°C with the indicated concentrations of ATP. Next, iDCs were extensively washed and co-cultured with autologous CD4^+ ^T cells that were previously pulsed with VSV-G-pseudotyped luciferase reporter viruses. Luciferase activity was assessed at 48 h following initiation of the co-culture. (C) CD4^+ ^T cells were infected with NL4-3Bal*env *either in absence or presence of ATP (400 μM). Cells treated with the antiviral compound efavirenz (EFV) were used as controls. Virus production was monitored by measuring the cell-free p24 contents over time. Data shown represent the means ± standard deviations of triplicate samples and are representative of three independent experiments.

We next set out to shed light on the mechanism underlying the ATP-mediated diminution in virus transfer. First the impact of ATP treatment on the stimulatory capacity of iDCs was evaluated in a mixed lymphocyte proliferation assay based on a previous report showing that ATP modulates the T-cell stimulatory capacity of DCs following a 48 h treatment [25]. Under our experimental conditions (i.e. treatment with ATP for 60 min), ATP had no effect on the stimulatory capacity of iDCs in a classical mixed lymphocyte reaction assay in which control or ATP-treated iDCs were cocultured with heterologous CD4^+ ^lymphocytes (Fig. 5A). It has also been demonstrated that ATP can induce a marked increase in expression of two genes encoding for negative regulators of T lymphocyte proliferation, namely thrombospondin-1 and indoleamine 2,3-dioxygenase [26]. To investigate the possibility that the release of such inhibitory mediators could affect HIV-1 expression in CD4^+ ^T cells, iDCs were incubated with ATP for 60 min, washed to remove ATP and co-cultured with autologous CD4^+ ^T cells previously pulsed with HIV-1-based reporter viruses pseudotyped with VSV-G. Data from this series of investigations reveal that virus gene expression in CD4^+ ^T cells is similar upon coculture with untreated or ATP-treated iDCs (Fig. 5B). Moreover, virus production in acutely infected CD4^+ ^T cells was not affected by a prior exposure to extracellular ATP (Fig. 5C). These results indicate that the ATP-mediated reduction in HIV-1 transmission is not related to any direct effect of extracellular ATP or ATP-treated iDCs on the process of virus infection in CD4^+ ^T cells. Thus ATP must act directly on iDCs and modulates the HIV-1 life cycle by affecting the level of viral entry, intracellular trafficking of virions within the endosomal apparatus and/or formation of the virological synapse.

**Figure 6:**
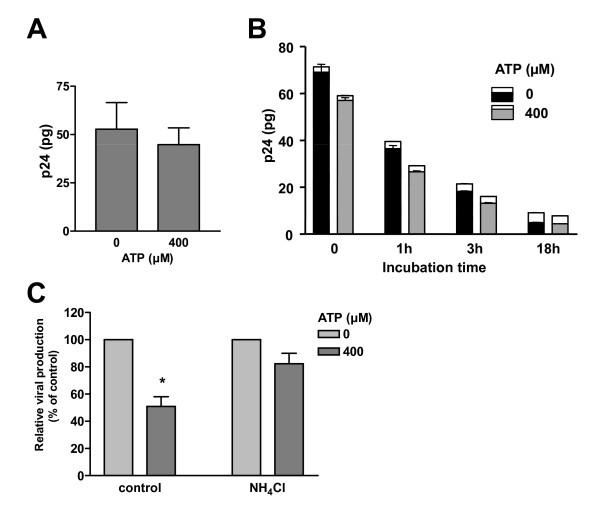
**ATP affects virus survival within iDC**. iDCs were initially pulsed for 60 min at 37°C with NL4-3Bal*env *in absence or presence of ATP (400 μM). Next, cells were washed extensively, treated with trypsin to remove uninternalized viruses and then either lysed immediately (A) or incubated at 37°C for the indicated time periods (B). The viral contents found either intracellularly and in cell-free supernatant were assessed using a p24 test. The data shown represent the means ± standard deviations of triplicate samples and are representative of three independent experiments. In panel B, the upper part of each bar represents p24 recovered in the supernatant, whereas the intracellular p24 content is shown in the lower part of each bar. (C) iDCs were either left untreated or pretreated with NH_4_Cl (20 mM) for 30 min. Next, iDCs were pulsed for 60 min at 37°C with NL4-3Bal*env *in absence or presence of the indicated concentrations of ATP. Cells were washed extensively to remove input virus and excess ATP and co-cultured with autologous CD4^+ ^T cells at a 1:3 ratio. Viral production was assessed two days following initiation of the co-culture. The data shown represent the means ± standard deviations calculated from four independent experiments with triplicate samples and are presented as relative viral production of ATP-treated cells compared to control cells for each condition. The asterisk denotes statistically significant data (*, P < 0.05).

It has been shown that HIV-1 can enter DCs either through an endocytic pathway or fusion at the plasma membrane following interaction with CD4 and CCR5. This latter mode of virus entry leads to the cytosolic delivery of viral material and results in productive infection, a process that is not affected by ATP. On the other hand, endocytosed viral particles can have two different fates, i.e. degradation within the endosomal apparatus or storage inside yet to be defined endocytic vacuoles followed by a subsequent transfer at the virological synapse. To analyze the effect of ATP on HIV-1 internalization, iDCs were pulsed with HIV-1 particles in absence or presence of ATP and, after removal of non-internalized particles by a trypsin treatment and several washes, the amount of cell-associated p24 was monitored. Results illustrated in Fig. 6A indicate that ATP causes a slight decrease in virus entry in iDCs (i.e. 16% in the experiment shown in Fig. 6A). Since the effect was variable and not consistently observed among the different DC preparations tested (i.e. ranging from 0 to 20%) (data not shown), we postulated that the ATP-mediated effect on HIV-1 transfer could be related to a very rapid degradation of internalized viruses during the trypsin treatment and washes. We then analyzed virus degradation and release over time. As shown in Fig. 6B, the difference in intracellular p24 contents between untreated and ATP-treated cells increases from 18% to 28% at the 1 and 3 h time points, thus suggesting a slightly higher rate of virus degradation in ATP-treated iDCs. It should be noted that the observed decrease in intracellular p24 is caused by degradation rather than by the release of viral particles since a very small amount of p24 was recovered in the medium, and this amount was similar in control and treated samples. To provide additional credence to the ATP-dependent effect on virus degradation, iDCs were first pretreated with NH_4_Cl, a lysomotropic weak base known to block endosomal acidification [30] and lysosomal degradation [31], and next used to transfer HIV-1 to autologous CD4^+ ^T cells. As demonstrated in Fig. 6C, the ATP-mediated diminution in HIV-1 transmission is drastically reduced upon treatment with NH_4_Cl (i.e. an average diminution of 50% in absence of NH_4_Cl compared to a decrease of 18% in the presence of NH_4_Cl for cells treated also with 400 μM of ATP). This observation suggests that ATP acts at least partly by inducing a more pronounced degradation of HIV-1 in acidified endosomes.

### ATP affects expression of some specific surface markers

**Figure 7:**
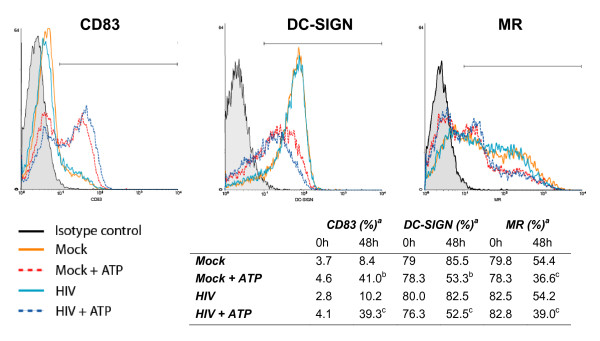
**Surface expression of CD83, DC-SIGN and MR is affected by ATP**. iDCs (5 × 10^5^) were either left untreated or treated for 60 min at 37°C with ATP (400 μM), NL4-3Bal*env *or ATP and NL4-3Bal*env*. Next, cells were extensively washed and either stained immediately or cultured for 48 h in complete medium before staining. Cells were stained with antibodies specific for CD83, DC-SIGN, or MR and analyzed by flow cytometry. Results from one representative experiment and flow data acquired after 48 h are presented. ^a ^%, Percentage of cells expressing the studied cell surface marker as defined by flow cytometry. Statistical analyses were performed on the results of three independent experiments (^b^, P < 0.01 ; ^c^, P < 0.05).

A chronic exposure of iDCs to ATP has been shown to induce a semi-maturation state characterized by an increased expression of activation markers such as CD54, CD80, CD83 and CD86 [22,25]. Previous findings indicate that HIV-1 can use several different attachment factors to interact with DCs such as the C-type lectin molecules DC-SIGN and MR. To analyze the consequences of a brief ATP exposure on expression of these cell surface markers, iDCs were either left untreated or treated with ATP and/or NL4-3Bal*env *for 60 min and were then thoroughly washed to remove ATP and/or viral particles. Membrane expression of CD83, DC-SIGN and MR was evaluated by flow cytometry either immediately following the 60 min treatment or 48 h later. Exposure to HIV-1 did not significantly affect the surface expression levels of any of the tested molecules at the two different time points tested. In contrast, a minor but reproducible increase in expression of the activation marker CD83 was induced very rapidly by ATP followed by a significant induction at the latest time point tested (Fig. 7). Interestingly, ATP was found to diminish expression of the two HIV-1 attachment factors DC-SIGN and MR but at the latest time point tested (i.e. 48 h). Other markers of DC maturation such as CD86 were also increased after ATP exposure (data not shown). These data indicate that exposure to extracellular ATP is sufficient to mediate profound changes in expression of cell surface constituents involved in the complex interplay between HIV-1 and iDCs.

## Discussion

Extracellular ATP that is released under inflammatory conditions is now recognized as a potent stimulus for the initiation of immune responses through activation of DCs. However, it may also play a role in preventing an exaggerated immune response by favouring an alternative DC maturation that skew the immune reaction towards either a Th2-type response or tolerance. Here we show that extracellular ATP may fulfill an additional unexpected role in the defence against infection by reducing HIV-1 transmission from iDCs to CD4^+ ^T cells.

Several lines of evidence indicate that ATP is affecting primarily HIV-1 *trans*-infection of CD4^+ ^T-cells (i.e. early transfer). First, the ATP-dependent reduction in virus transfer is not modified when viral replication in iDC is inhibited by an antiretroviral compound such as efavirenz. Second, ATP treatment has no effect whatsoever on HIV-1 replication in iDCs in the absence of CD4^+ ^T cells. Third, the transfer of both X4- and R5-tropic viruses is affected by ATP and it is known that replication of X4-using virions in DCs is very inefficient [7,32]. Indeed previous studies have reported that X4 viruses do replicate poorly and slowly in DCs and that only replicative viruses are efficiently transferred few days after pulsing [14,16]. This could provide an explanation for the ATP-mediated diminution in virus transfer that can be seen only in the first 2–3 days following initiation of the co-culture. At later time points, the detected viruses are newly formed virions arising from replication within iDCs, which is not affected by ATP.

Treatment of DCs with ATP has been shown previously to induce up-regulation of two genes involved in immunosuppression, i.e thrombospondin-1 and indoleamine 2,3 dioxygenase [26]. It would then be plausible that such mediators could impair HIV-1 infection of CD4^+ ^T cells in DC-T cell cocultures through an anti-proliferative effect, leading to a decreased viral production. However, such a mechanism is very unlikely since we demonstrate that virus replication in autologous CD4^+ ^T cells is not affected upon co-culture with ATP-treated iDCs (Fig. 5). Moreover, ATP treatment resulted in a similar effect on HIV-1 transfer from iDCs to TZM-bl, a HeLa-derived indicator cell line that is not sensitive to such inhibitors of cell proliferation (data not shown). Therefore, it can be proposed that ATP influences HIV-1 transfer through a possible effect on the complex interactions between iDCs and HIV-1 such as binding and internalization, survival within endosomal compartments and/or creation of the virological synapse. Additional experiments demonstrate that ATP induces a slight decrease in the process of HIV-1 internalization (Fig. 6), but this effect is too weak and inconsistent to explain the more dramatic reduction in virus transfer. Hence, ATP must affect HIV-1 survival inside iDCs and/or the signal that triggers movement of the virus-containing organelles towards the infectious synapse. Treatment with a weak base such as NH_4_Cl that inhibits endosomal acidification greatly reduced the effects of ATP, therefore suggesting that the ATP-mediated signal transduction pathway either target viruses in different, more acidic endosomal compartments or directly induce acidification of HIV-1-containing intracellular organelles. Indeed, endosomal acidification by a proton translocating ATPase has been shown to be regulated via a Gi-mediated translocation pathway [33], which is activated by P2Y receptors upon ATP stimulation. Treatment with NH_4_Cl or chloroquine, another lysosomotropic agent, seems to have a more dramatic effect in Raji-DC-SIGN cells where it completely abolishes transfer at low viral titers [8]. This confirms that transfer mechanisms are quite different in that more artificial model system. In Raji-DC-SIGN cells, viruses are transferred exclusively through DC-SIGN binding and only DC-bound viruses are transferred, whereas several studies suggest that in human primary iDCs internalized viruses are transferred [7-11] and not exclusively through DC-SIGN binding [5,16]. These discrepancies indicate that observations made in cell lines should be interpreted with caution, as they do not necessarily apply to human primary cells. Besides, several observations suggest that ATP could influence the intracellular trafficking in DCs. For example, stimulation with ATP or other agonists of the P2X_7 _receptor induces the release of microvesicles containing various proteins such as caspases, cathepsin D and the procoagulant tissue factor [34,35]. Moreover, ATP has been reported to modify the DC endocytic activity [25] as well as to induce actin polymerization and chemotaxis in DCs [28]. Surprisingly, a recent report suggests that HIV-1 transmission is primarily due to the transfer of DC surface-bound virions to CD4^+ ^T cells [6]. However, other studies are more in accordance with the importance of intracellular trafficking in the transfer. For example monensin, an antibiotic that disrupts the Golgi apparatus and inhibits vesicular transport, was shown to decrease DC-mediated HIV-1 transmission [10]. Although our data with NH_4_Cl strongly suggest that, at least under our experimental conditions, ATP affects the transfer of internalized virions, we cannot completely rule out the possibility that ATP could also impair transmission of surface-bound virions, possibly by interfering with the clustering of lipid rafts.

Our observation that extracellular ATP affects HIV-1 transfer in iDCs, but not in mDCs, deserves a closer attention. Since HIV-1 cannot productively infect mDCs [36], this suggests that only viruses that are stored within intracellular organelles are transferred, similarly to the first phase in iDCs which is affected by ATP. Interestingly, ATP appears to induce profound modifications in iDCs but seems to have little effect on mDCs. For example, although levels of mRNAs coding for P2X and P2Y receptors do not change during DC maturation, P2YR agonists were found to act as chemotactic stimuli only in iDCs [28]. It is very unlikely that the observed ATP-driven reduction in virus transfer would be due to the previously reported semi-maturation state induced by ATP [22,23] since mDCs have been shown to efficiently transfer HIV-1. There might be profound differences in the signalling induced by ATP in iDCs versus mDCs. Additionally, the mechanisms responsible for HIV-1 storage and transfer are quite different between iDCs and mDCs. Indeed, it has been shown that mDCs will propagate HIV-1 much more efficiently than iDC, and independently of C-type lectins [10,37]. In addition, HIV-1 tends to accumulate in late endosomal compartments, close to the cell membrane in iDCs, whereas viral particles are found in large complex vesicles in mDCs, where they colocalize with tetraspanins CD9, CD63 and CD81 [37-39]. Typically DC-SIGN acts as an antigen receptor in iDCs and DC-SIGN/ligand complexes are directed toward lysosomal compartments [40]. The virus itself seems to be diverted from this pathway and HIV-1/DC-SIGN complexes are directed to non-lysosomal acidic organelles [8]. The exact mechanism(s) that governs this escape from lysosomal degradation is still unclear but it is very likely that the ATP-mediated biochemical events will modify this process by favouring virus degradation. The fact that transfer from mDCs, which does not depend on DC-SIGN, is not affected by ATP support this hypothesis. Incidentally, the transport of HIV-1 to the proteasome in DCs is mediated by the interaction between DC-SIGN and the leukocyte-specific protein 1 (LSP-1). In fact, it has been shown that down regulation of LSP-1 will enhance HIV-1 transfer due to a decreased virus degradation in the proteasome [15]. This actin-binding cytosqueletal protein is regulated through phosphorylation by various kinases including protein kinase C and mitogen-activated protein kinase-activated protein kinase 2 [41,42]. It is possible that the ATP-mediated signal transduction events might interfere with this regulation and enhance LSP-1 activity, leading to an increased HIV-1 degradation in the proteasome with an ensuing decrease in virus transfer.

Another indication that ATP might be affecting the endosomal recycling process is the small but consistent increase in CD83 expression that can be seen immediately following exposure of iDCs to ATP. Although not expressed at the surface of iDCs, CD83 is preformed and can be detected in perinuclear regions [43]. A previous study has demonstrated that surface expression of CD83 in iDCs can be induced by inhibition of endocytosis [44]. It can thus be proposed that the ATP-mediated signals can modify the recycling process leading to an increase in CD83 surface expression but also to a decrease in viral transfer. Conversely, extracellular ATP induces a decrease in DC-SIGN expression. This decrease is too slow to have any effect on HIV-1 internalization and in fact the importance of DC-SIGN for virus entry has been recently questioned [13]. Indeed, the inconsistent and minor reduction in HIV-1 internalization in the presence of ATP is too modest to account for the decrease in transfer efficiency. The importance of DC-SIGN at later time points has been controversial. Although some reports indicate an absence of the DC-SIGN molecule at the DC-CD4^+ ^T cell interface [9], other studies indicate that DC-SIGN is required for the formation of the infectious synapse (but not the formation of DC-T cell conjugates) and indeed DC-SIGN knock-down inhibits virus transmission by iDCs [13]. Hence, a diminished DC-SIGN expression following ATP exposure could lead to a reduced number of DC-T cell synapses, leading to a lower number of infected CD4^+ ^T cell (Fig. 3). A recent study has shown that DC-SIGN is important only for the transfer of non-internalized virus, whereas the galactosyl ceramide receptor would be crucial for the transfer of internalized viruses [5]. It would be of great interest to analyze the effect of extracellular ATP on the expression and function of this receptor. Although ATP is also inducing a reduced expression of MR at 48 h following treatment, the precise contribution of MR in the observed reduction in HIV-1 transfer is still undefined given the paucity of data with respect to the involvement of this C-type lectin receptor in some basic functions of DCs such as endocytosis and antigen processing and presentation.

Another possible event that could be affected by ATP is the formation of the virological synapse. Before the contact between DCs and CD4^+ ^T cells, virus particles are evenly distributed within DCs. However, upon a contact with CD4^+ ^T lymphocytes, viruses very rapidly become concentrated at the DC-T cell interface [9]. This observation suggests that a signal is sent within DCs upon a contact with CD4^+ ^T cells, which triggers the formation of an infectious synapse and directs HIV-1 trafficking towards that synapse. It is then possible that ATP will affect that triggering signal, then inhibiting the formation of a functional synapse. Further analyses are required to identify the nature of that signal, how it triggers the movement of virus-containing organelles towards the infectious synapse, and how exposure to extracellular ATP can possibly interfere with this mechanism.

## Conclusion

The ATP-mediated effect on viral transfer reveals obvious implications for HIV-1 pathogenesis because DCs are not only the first target cells following mucosal invasion with HIV-1 but they are also the first line of defence against such invasion. The primary role of these potent antigen presenting cells as peripheral sentinels is to induce an efficient immune response against pathogens. Extracellular ATP, by interfering with HIV-1 transfer to CD4^+ ^T cells and promoting virus degradation within iDCs, might favour the processing and presentation of viral antigenic peptides that will lead to a specific antiviral response. The other well-known effects of ATP on DC biology, i.e. cell activation and migration towards lymph nodes, might accentuate further the HIV-1 specific immune response. Because ATP is released from activated T lymphocytes, activated platelets, or necrotic cells, its presence is a hallmark of inflamed tissues. Indeed, with an intracellular concentration of ATP ranging from 5 to 10 mM, any plasma membrane damage will promote a massive release of ATP. Hence, although extracellular ATP concentrations have been estimated to be in the low micromolar range due to a rapid hydrolysis by ectonucleases [45], it is believed that in inflamed tissues, the massive ATP release coupled with a down-regulation of the ecto-ATPase CD39 may lead to an accumulation of ATP which can reach very high concentrations in protected compartments [46]. Moreover, in vivo studies have provided clear evidence for sustained ATP release at sites of tissue injury [47,48]. The concentrations used in our studies can then be easily attained in situations of tissue damage and inflammation. Our results showing a reduced HIV-1 transfer from iDCs to autologous CD4^+ ^T cells represent a new functional role for ATP that is already considered as a constitutive danger signal for the immune system.

## Methods

### Reagents

ATP, phytohemagglutinin-L (PHA-L) and lipopolysaccharide (LPS) were purchased from Sigma (St-Louis, MO). Interleukin-2 (IL-2) and efavirenz were obtained through the AIDS Repository Reagent Program (Germantown, MD). Interferon-gamma (IFN-γ) and IL-4 were purchased from R&D Systems (Minneapolis, MN), whereas GM-CSF was a generous gift from Cangene (Winnipeg, MAN). The culture medium consisted of RPMI 1640 supplemented with 10% fetal bovine serum, penicillin G (100 U/ml), streptomycin (100 U/ml) and glutamine (2 mM), which were all purchased from Wisent (St-Bruno, QC).

### Antibodies

The anti-CD3 OKT3 hybridoma (specific for the ζ chain of the CD3 complex) was obtained from the American Type Culture Collection (Manassas, VA). The anti-CD19 (clone LT19) and anti-CD14 (clone MEM-18) were obtained from EXBIO Praha (Prague, Czech Republic) while the anti-CD83 (clone HP15E) was purchased from Research Diagnostics (Concord, MA). The FITC-conjugated anti-DC-SIGN (clone eB-h209) and anti-CD14 (clone 61D3) were purchased from *e*Bioscience (San Diego, CA). Phycoerythin (PE)-conjugated and FITC-conjugated goat anti-mouse immunoglobulin G (IgG) were obtained from Jackson ImmunoResearch Laboratories (West Grove, PA).

### Cells

PBMCs were prepared by centrifugation on a Ficoll-Hypaque density gradient as described previously [49,50]. Next, CD14^+ ^cells (i.e. monocytes) were isolated by using a positive selection kit according to the manufacturer's instructions (MACS CD14 micro beads from StemCell Technologies, Vancouver, BC). The CD14^+ ^cells were cultured in six-well plates at a density of 10^6 ^cells/ml. To generate iDCs, purified monocytes were cultured in complete culture medium that was supplemented every other day with GM-CSF (1,000 U/ml) and IL-4 (200 U/ml) for 7 days, and the maturation of DCs was induced on the fifth day by culturing them for 48 h with the above-described cytokines supplemented with IFN-γ (1,000 U/ml) and LPS (100 ng/ml) as described previously [18,29]. Expression of CD3 and CD19 was measured to assess contamination with T and B cells, respectively. Autologous CD4^+ ^T cells were isolated using a negative selection kit according to the manufacturer's instructions (StemCell Technologies), activated with PHA-L (1 μg/ml) and maintained in complete culture medium supplemented with IL-2 (30 U/ml) at a density of 2 × 10^6 ^cells/ml. Experiments were performed with cell preparations that were highly enriched in the studied cell subpopulations (e.g. DCs: purity >95%; CD4^+ ^T cells: purity >98%).

### Production of virus stocks

Virions were produced by transient transfection in human embryonic kidney 293T cells as previously described [51]. Plasmids used include pNL4-3 (X4-tropic), pNLHSA-IRES (X4-tropic), pJR-CSF (R5-tropic), pNL4-3Bal*env *(R5-tropic), pNL4-3*Luc*^+^*E*^-^*R*^+^, pcDNA-1/Amp-based JR-FL envelope (R5-tropic) and pHCMV-G. The pNL4-3Bal*env *vector (provided by R. Pomerantz, Thomas Jefferson University, Philadelphia, PA) was generated by replacing the *env *gene of the T-tropic HIV-1 strain, NL4-3, with that of the macrophage-tropic HIV-1 Bal strain, thus resulting in an infectious molecular clone with macrophage-tropic properties [52]. The pNLHSA-IRES molecular construct was obtained by replacing the *eGFP *gene in the NLENG1-IRES vector (NL4-3 backbone) [53] with the coding sequence for mouse heat stable antigen (HSA) (Imbeault et al., manuscript in preparation). The luciferase-encoding pNL4-3*Luc*^+^*E*^-^*R*^+ ^and the R5-tropic envelope-encoding pcDNA-1/Amp-based JR-FL envelope vectors were kindly provided by N. R. Landau (The Salk Institute for Biological Studies, San Diego, CA) and were used to generate single-cycle luciferase reporter viruses. The other molecular constructs and HIV-1 strains were obtained through the AIDS Repository Reagent Program. The pHCMV-G plasmid codes for the broad host-range vesicular stomatitis virus envelope glycoprotein G (VSV-G) and is placed under the control of the human cytomegalovirus promoter. HIV-1-based reporter viruses pseudotyped with VSV-G were produced upon cotransfection of 293T cells with pNL4-3*Luc*^+^*E*^-^*R*^+ ^and pHCMV-G. Progeny viruses were also produced upon acute infection of PBMCs and purified CD4^+ ^T cells for 7 days with two laboratory strains of HIV-1 (i.e. JR-CSF and NL4-3Bal*env*) and the X4-tropic clinical isolate 92HT599. The virus-containing supernatants were filtered through a 0.22 μm cellulose acetate syringe filter and normalized for virion content using an in-house sensitive double-antibody sandwich enzyme-linked immunosorbent assay (ELISA) specific for the viral p24 protein [54].

### Virus transfer studies

DCs (3.3 × 10^4 ^cells in 100 μl) were pulsed with the studied virus preparations (10 ng of p24^*gag*^/10^5 ^cells) for 60 min at 37°C in absence or presence of various concentrations of ATP. Next, the virus-cell mixture was washed three times with phosphate-buffered saline (PBS) to remove input virus. iDCs and mDCs were co-cultured with autologous activated CD4^+ ^T lymphocytes (ratio 1:3) in complete RPMI medium supplemented with IL-2 (30 U/ml) in 96-well plates in a final volume of 200 μl. Every two days, half of the medium was removed and kept frozen at -20°C and fresh medium was added to the culture. Virus production was estimated by measuring the p24 levels in cell-free culture supernatants. Infection with luciferase-encoding viruses was assessed 3 days following initiation of the co-culture by measuring luciferase activity. Transfer studies with NLHSA-IRES viruses were performed the same way but using 3 × 10^5 ^iDC and 10^6 ^CD4^+ ^T cells per sample. Mock-infected cells were used as controls. Three days after initiation of the co-culture, cells were stained either with an isotype-matched irrelevant control antibody (i.e. IgG_1_) or anti-CD3 followed by an FITC-conjugated goat anti-mouse IgG and a biotin-conjugated anti-HSA followed by R-PE-labeled streptavidin. Stained cells were fixed with 2% paraformaldehyde for 30 min at 4°C and then analyzed by flow cytometry (Epics ELITE ESP; Coulter Electronics, Burlington, ON). Single stained cells were also used as controls for compensation adjustments.

### Virus infection of iDCs and CD4^+ ^T cells

*De novo *virus production in iDCs was monitored by incubating such cells (5 × 10^5^) with HIV-1 (50 ng of p24^*gag*^) for 60 min at 37°C in absence or presence of ATP. After three washes with PBS, the cells were maintained in complete culture medium supplemented with GM-CSF (1000 U/ml) and IL-4 (200 U/ml). Purified CD4^+ ^T cells were incubated for 2 h with NL4-3Bal*env *(10 ng p24^*gag*^/10^5 ^cells) in absence or presence of ATP. After three washes with PBS, the cells were cultured in complete culture medium supplemented with IL-2 (30 U/ml). Virus production was estimated by assessing the p24 levels in cell-free culture supernatants.

### Virus entry and degradation assays

iDCs (10^5 ^cells) were incubated with NL4-3Bal*env *(10 ng p24^*gag*^) in absence or presence of ATP for 60 min at 37°C. Cells were then washed once with PBS, treated with trypsin for 5 min at 37°C to remove all uninternalized virions, washed three times and then either immediately lysed or incubated in 100 μl complete RPMI for 0, 1, 3, or 18 h. Cells were then separated from medium and the p24 contents were evaluated in both cells and cell-free culture supernatants.

### Statistical analysis

Statistical analyses were performed according to methods outlined in Zar [55]. Means were compared using either the Student's test or single factor ANOVA followed by Dunnett's multiple comparison when more than two means were considered. Results of three or four experiments were always used for these analyses even when results of one representative experiment were presented in a Figure. Because infection levels vary largely among various DC preparations, a repeated measures ANOVA test had to be used. P-values of less than 0.05 were considered statistically significant. The GraphPad InStat version 3.05 software (GraphPad Software, Inc) was used for all analyses.

## Competing interests

The author(s) declare that they have no competing interests.

## Authors' contributions

CB designed and carried out all the experiments, and drafted the original manuscript. CG participated in the design and interpretation of most experiments, provided some of the cells and virus stocks, and helped to draft the manuscript. MI designed and constructed the NLHSA-IRES vector. MJT supervised and coordinated the study and finalized the manuscript. All authors read and approved the final manuscript.
